# Preoperative inferior vena cava ultrasound for volume optimization in resection of functioning pheochromocytoma and paraganglioma: association with perioperative adverse outcomes in a prospective observational cohort

**DOI:** 10.3389/fendo.2026.1844538

**Published:** 2026-07-23

**Authors:** Jiali Tang, Yuelun Zhang, Dan Wu, Xiang Shi, Yuguang Huang

**Affiliations:** 1 Department of Anesthesiology, Peking Union Medical College Hospital, Peking Union Medical College & Chinese Academy of Medical Sciences, Beijing, China; 2 Institute of Clinical Medicine, Peking Union Medical College Hospital, Peking Union Medical College & Chinese Academy of Medical Sciences, Beijing, China; 3 Department of Anesthesiology, Guang’an People’s Hospital, Guang’an, China; 4 Operating Room, Peking Union Medical College Hospital, Peking Union Medical College & Chinese Academy of Medical Sciences, Beijing, China

**Keywords:** inferior vena cava ultrasound, perioperative adverse outcome, pheochromocytomas and paraganglioma, point-of-care ultrasound, preoperative volume, α-blockade treatment

## Abstract

**Background:**

Optimizing intravascular volume (IV) before surgery is crucial for patients undergoing resection of functioning pheochromocytomas and paragangliomas (PPGLs). While point-of-care ultrasound (POCU) is a noninvasive technique to assess IV, the use of POCU to evaluate preoperative IV and its relationship to perioperative adverse outcomes (PAOs) remains largely unexplored.

**Methods:**

We conducted a prospective cohort study including patients who underwent PPGL resection following preoperative α-blockade. Preoperative IV was assessed using inferior vena cava (IVC) ultrasound and clinical parameters. We used logistic regression models to examine the associations between preoperative IV and intraoperative hemodynamic instability (IHI), perioperative organ dysfunction (POD), and PAO. We also compared the predicting abilities of IVC ultrasound measurements and clinical parameters for PAOs.

**Results:**

A total of 80 patients were recruited and analyzed. The study population carried a heavy comorbidity burden (median age-adjusted Charlson Comorbidity Index 4), thus the median duration of preoperative α-blockade was 60 (30, 98) days. Based on IVC ultrasound, 27 patients were classified as having appropriate volume status and 53 as inappropriate volume status. We found no significant association between IVC ultrasound assessed IV status and IHI, POD, and PAO. Furthermore, neither IVC ultrasound assessments nor clinical parameters could discriminate PAOs, and the predictive performance of the two methods did not differ significantly. However, this finding should be interpreted with caution, as the study was likely to be underpowered (enrolled 80 of 364 planned patients, 22%).

**Conclusion:**

In this study with a sample size of 80 patients, no independent association was found between preoperative IVC ultrasound measurements and PAOs in patients undergoing PPGL resection with an extended duration of preoperative α-blockade preparation [60 (30, 98) days]. The utility of point-of-care IVC ultrasound in guiding preoperative volume optimization for these patients merits further exploration.

## 1 Introduction

Pheochromocytomas and paraganglioma (PPGL), rare neuroendocrine tumors, pose significant surgical and anesthetic challenges due to the potential for massive intraoperative catecholamine release and life-threatening cardiovascular complications. Mortality associated with PPGL surgery has declined due to advances in perioperative management, anesthesia and surgical techniques (1–5). Nevertheless, preoperative meticulous attention to arterial pressure control and volume optimization remains the non-negotiable cornerstones of outcome optimization.

The standard preoperative regimen for PPGL resection surgery, as recommended by guidelines, involves α-adrenergic blockade coupled with a high-sodium diet and increased fluid intake for at least 14 days. This strategy targets the normalization of blood pressure and heart rate and the blunting of catecholamine-mediated cardiomyopathy. However, a crucial, and frequently overlooked, benefit of this regimen is its ability to restore contracted intravascular volume (IV) (6–8). Restoration of adequate IV is considered critical to prevent severe perioperative hypotension, a common complication after tumor resection associated with the occurrence of postoperative organ dysfunction (9). Intraoperative goal-directed fluid therapy (GDFT) is recommended for anesthetic management. Abundant literature indicates that GDFT reduces postoperative complications, shortens the length of hospital stay, and promotes postoperative functional recovery (10–14). It was reported that transesophageal echocardiography (TEE) and esophageal Doppler were used to guide fluid therapy during PPGL resection surgery (15, 16). Currently, the evaluation of preoperative IV is still based on indirect clinical parameters, such as blood pressure, heart rate, weight, HCT value, and symptoms of orthostatic hypotension and nasal congestion. However, these indirect indicators may not be primarily determined by IV, and impacted by heart function, vascular resistance, and extravascular volume (17). Therefore, more reliable assessments are required to directly evaluate preoperative IV and guide preoperative volume optimization.

Ultrasound is a noninvasive point-of-care tool increasingly used by anesthesiologists for perioperative cardiovascular assessment (18). Advantages include a documented relationship between IVC ultrasound measurements (including IVC diameter and collapsibility index) and right atrial pressure, easy accessibility of ultrasound equipment, relatively straightforward image acquisition, and good reproducibility (19, 20). Inferior vena cava (IVC) diameter measurements with respiration have been recommended as a rapid and noninvasive method for estimating IV status (21). Currently, it plays a role in predicting volume responsiveness in various clinical settings (22–24), and hypotension following induction of general or spinal anesthesia (25–27), which could potentially identify high-risk patients and guide treatment strategies.

Therefore, in this prospective cohort study we aimed to investigate the association of IVC ultrasound assessed preoperative IV and perioperative adverse outcome, and to compare the predictive capacity of IVC ultrasound assessed IV with that of clinical parameters for perioperative adverse outcome.

## 2 Materials and methods

### 2.1 Study design and setting

This was a prospective, observational cohort study conducted at a tertiary academic care center in Beijing from June 2022 to July 2024. The study protocol was prospectively registered with the Chinese Clinical Trial Registry (ChiCTR2200060382, https://www.chictr.org.cn/showprojEN.html?proj=171280) and approved by the Ethics Committee (JS-3543). The protocol was not published in a journal. The study was conducted adhered to the Declaration of Helsinki World Medical Association. Informed consent was obtained from all patients. The study is reported according to the Strengthening the Reporting of Observational Studies in Epidemiology (STROBE) guidelines (**Supplementary Table 1**) (28).

### 2.2 Study population

Eligible participants were adult hypertensive patients (aged 18–80 years) undergoing functioning PPGL resection who had received preoperative α-blockade. Exclusion criteria included: (1) refusal to participate; (2) inability to cooperate; (3) unsuccessful tumor resection, or converted to open surgery; (4) previous PPGL surgery; (5) inability to obtain satisfactory ultrasonographic images of the subxiphoid IVC; (6) conditions affecting IVC pressure, such as constrictive pericarditis, pneumothorax, or severe cardiac disease; and (7) postoperative pathology inconsistent with PPGL. Patients were followed up for 30 days postoperatively.

### 2.3 Protocol of preoperative preparation

All patients received α-blockade preoperatively for at least 2 weeks. Meanwhile, a diet with high-sodium and free fluid intake were suggested during α-blockade treatment. Blood pressure (BP) and heart rate (HR) in supine and upright position were monitored at home three times a day. Patients were followed up regularly at outpatient clinics, while the dose of α-blockade was adjusted according to BP and HR. The adequate preoperative preparation has been considered, when hypermetabolic syndrome, catecholamine-induced cardiomyopathy, abnormal glucose metabolism, arrhythmias, sustained malignant hypertension, paroxysmal hypertension, and orthostatic hypotension were effectively under control (7, 8, 29). Afterwards, patients were admitted to the hospital for PPGL resection surgery.

### 2.4 Assessment of preoperative intravascular volume using IVC ultrasound

IVC ultrasound was performed one day prior to surgery, following methods previously described. Briefly, a phased array transducer (1–5 MHz, X-Porte, SonoSite, Bothell, WA, USA) was positioned subxiphoidally to obtain a longitudinal view of the IVC with the patient supine. The IVC diameter was measured 2 cm caudal to its junction with the right atrium. Patients were asked to take deep breaths, and the minimal and maximal IVC diameters were recorded at the end of inspiration (IVC_min_) and expiration (IVC_max_). The collapsibility index (cIVC) was calculated as: cIVC = (IVC_max_ - IVC_min_)/IVC_max_. Measurements were obtained over three respiratory cycles, and average values were calculated.

The relationship between IVC measurements and right atrial (RA) pressure has been comprehensively established by ASE/EACVI guidelines (19): an IVC diameter < 2.1 cm with inspiratory collapses >50% suggests an RA pressure of 3 mm Hg (range, 0–5 mm Hg), whereas an IVC diameter > 2.1 cm with collapses < 50% suggests an elevated RA pressure of 15 mm Hg (range, 10–20 mm Hg). For scenarios in which IVC diameter and collapse do not fit this paradigm, an intermediate RA pressure of 8 mm Hg (range, 5–10 mm Hg) may be assigned.

However, the classification of preoperative volume status in this study was specifically adapted to the unique hemodynamic context of PPGL patients undergoing resection. These patients routinely receive aggressive preoperative volume expansion (high-salt diet, liberal fluid intake) to counteract catecholamine-induced vasoconstriction and intravascular depletion. In this context, an IVC profile consistent with “normal” RA pressure indicates failure to achieve the intended preload expansion, rather than true euvolemia. Therefore, inadequate volume status was defined as IVC diameter ≤ 2.1 cm with collapse > 50%.

In summary, patients were categorized into two cohorts: appropriate volume (AV) or inappropriate volume cohort (IAV). Appropriate volume was defined as IVC diameter ≤ 2.1 cm with cIVC < 50%, or IVC diameter > 2.1 cm with cIVC > 50%. Inappropriate volume status was defined as IVC diameter ≤ 2.1 cm with cIVC > 50% (indicating hypovolemia), or IVC diameter > 2.1 cm with cIVC < 50% (indicating hypervolemia). These criteria were finalized before the recruitment of patients. The surgical team, anesthesiologists, patients, and outcome assessors were blinded to IVC measurements. Surgical eligibility was independent of IVC measurements.

### 2.5 Assessment of preoperative intravascular volume using clinical parameters

Clinical parameters were symptoms (orthostatic hypotension, and nasal congestion) and physical signs (upright systolic blood pressure, weight gain, decrease in hematocrit) observed or reported 2 to 3 days preceding surgery. Orthostatic hypotension was defined as a decrease in systolic blood pressure (SBP) of ≥ 20 mmHg or a decrease in diastolic blood pressure of ≥ 10 mmHg within 3 minutes of standing, compared to measurements obtained after a period of at least 5 minutes of rest in the supine position. Upright SBP was measured at the same time. Weight gain was defined as an increase in body weight compared to the pre-α-blockade baseline weight. Decrease in hematocrit (HCT) was defined as a reduction in HCT from the baseline value (measured prior to α-blockade treatment) to HCT value obtained preoperatively. Nasal congestion was defined as the patient’s subjective report of nasal congestion symptoms.

Based on these clinical parameters, patients were considered to have inappropriate IV status if any of the following criteria were met: presence of orthostatic hypotension, absent weight gain, absent decreased HCT, lack of nasal congestion, upright SBP < 90 mmHg. Patients not meeting any of these criteria were defined as having appropriate IV status.

### 2.6 Protocol of anesthesia and surgery management

A standardized anesthetic and surgical approach were implemented for all patients undergoing PPGL resection. General anesthesia was induced with intravenous midazolam (1–2 mg), propofol (2–3 mg/kg), either fentanyl (1-2 μg/kg) or sulfentanyl (0.1-0.2 μg/kg), and rocuronium (0.6-1mg/kg) followed by tracheal intubation. Anesthesia was maintained with sevoflurane (0.8-1.3 MAC) in an air−oxygen mixture. The bispectral index was continuously monitored, with titration of anesthetic agents to maintain a target range of 40-60. Invasive arterial blood pressure monitoring and central venous access were established. Intraoperative analgesia and neuromuscular blockade were maintained with intermittent boluses of opioids and rocuronium, respectively, as clinically indicated. Fluid management was guided by pulse pressure variation, with a target range of 8-12%. Vasoactive support was provided as needed: norepinephrine and/or epinephrine for hypotension, and phentolamine and/or nitroprusside for hypertension, targeting hemodynamic parameters within 20% of baseline values. Patients underwent standardized laparoscopic resection of PPGL performed by surgeons with more than 10 years of operative experience.

### 2.7 Protocol of management in intensive care union and surgical ward

After surgery, patients were transferred to the intensive care union (ICU) with endotracheal tube. In ICU, the organ function and electrolyte homeostasis were monitored and managed. Chest X-ray and blood tests (for hepatic and renal function) were routinely performed, while computed tomography, electrocardiogram, myocardial enzyme test and other laboratory examination were performed as appropriate. After the trachea had been extubated and organ functions and electrolyte homeostasis were stable, the patient was transferred from the ICU to the surgical ward.

### 2.8 Definitions of perioperative adverse outcomes

In the present study, perioperative adverse outcome was a composite of intraoperative hemodynamic instability and postoperative organ dysfunction. The primary outcome of interest was this composite perioperative adverse outcome. Data were independently collected by research staff blinded to patient cohort, clinical care, via in-person interviews, electronic medical record review, and telephone follow-up as appropriate.

Intraoperative hemodynamic instability was defined as either sustained hypertension or sustained hypotension during surgery. Sustained hypertension was characterized by a SBP > 160 mmHg lasting more than 10 minutes. Sustained hypotension was defined as SBP < 80 mmHg lasting more than 10 minutes. Postoperative organ dysfunction included pulmonary, hepatic, renal function impairments, vascular events, and surgical site infections within 30 days post-surgery, as follows: (1) pulmonary dysfunction: delayed extubating (> 24 hours or reintubation after extubating), pneumonia, acute lung injury, acute respiratory distress syndrome, atelectasis, bronchospasm, pulmonary edema and other conditions requiring prolonged oxygen therapy; (2) Hepatic dysfunction: for patients with normal preoperative liver function, total bilirubin (TBil) > 22.2 μmol/L, alkaline phosphatase (ALP) > 125 U/L, alanine aminotransferase (ALT) > 50 U/L, or aspartate aminotransferase (AST) > 40 U/L; for patients with pre-existing liver abnormalities, any 50% increase in TBil, ALP, ALT, or AST compared to the most recent preoperative values; (3) renal dysfunction: for patients with normal preoperative serum creatinine, creatinine > 84 μmol/L (female) or 104 μmol/L (male); for patients with pre-existing renal abnormalities, a 50% increase in serum creatinine from the most recent preoperative test; (4) vascular events: including arrhythmias, acute coronary syndrome, elevated high-sensitivity cardiac troponin (hs-cTnI) (> 54 ng/L), stroke, pulmonary embolism, etc.; (5) surgical site infection: including superficial and deep surgical site infection as well as organ/space infection.

### 2.9 Statistical analysis

Continuous variables were presented as mean ± standard deviation or median (interquartile range (IQR)) based on distribution. Categorical variables were expressed as numbers and percentages. To evaluate the associations between parameters estimating preoperative IV and perioperative outcomes, odds ratios (ORs) with 95% confidence intervals (CIs) were calculated using binary logistic regression with both unadjusted and adjusted for clinically relevant covariates. Based on literatures and clinical experience, the following covariates were included in the adjusted models: age, sex, tumor size, plasma metanephrine concentration, plasma normetanephrine concentration, age-adjusted Charlson Comorbidity Index (ACCI), and Assess Respiratory Risk in Surgical Patients in Catalonia (ARISCAT) score. Weighted kappa statistics were used to examine the agreement between IVC ultrasound measurements and clinical parameters in assessing preoperative IV status. The ability of preoperative parameters estimating IV status for predicting perioperative outcomes was assessed using receiver-operating-characteristic (ROC) curve analysis. The areas under the ROC curves (AUCs) were compared between IVC ultrasound measurements and clinical parameters for predicting PAOs.

Sub-group analyses were not planed. Sensitivity analyses were performed. First, to address potential small-sample bias and data sparsity, a sensitivity analysis was performed using Firth’s penalized logistic regression. The second sensitivity analysis was conducted to examine whether our finding was robust to the choice of cut-offs of IVC ultrasound measurements, we modeled both two IVC diameters (IVC_max_ and IVC_min_) and collapsibility index (cIVC) as continuous variables using restricted cubic splines with 3 knots (10th, 50th and 90th percentiles) in logistic regression to reveal the potential non-liner relationship. The median value was chosen as the reference. Non-linearity was examined by a likelihood-ratio test comparing the spline model with the linear model. Although preoperative volume status is assessed in a binary fashion in clinical practice (based on the presence or absence of orthostatic hypotension, weight gain, decreased HCT, and nasal congestion), the arbitrary categorization of continuous variables results in a loss of statistical power. Thus, the third sensitivity analysis was performed by treating IVC measurements (IVC_max_, IVC_min_, cIVC), upright SBP, gain weight and HCT alternation as continuous variables rather than dichotomous variables using Firth logistic regression. Additionally, adjusted Firth logistic regression was performed to evaluate the associations of three IVC measurements with perioperative outcomes. The last sensitivity analysis was conducted to examine the relationship of IVC ultrasound assessed IV status and counts of perioperative adverse outcomes. Poisson regression model with robust variance estimation was used.

Based on the assumption of a 33.7% PAOs incidence in the AV group and a 48.4% in the IAV group (23), a two-sided α level of 0.05, a power (1-β) of 0.8, and an estimated dropout rate of 5%, a sample size of 182 patients per group (364 total) was indicated. The sample size was calculated using PASS software.

Statistical significance was defined as a two-sided p-value < 0.05. Missing data for the postoperative organ dysfunction within 30 days post-surgery would be addressed through multiple imputation after checking the missing mechanism. Statistical analyses were performed using IBM SPSS Statistics software, version 29.0, while Firth logistic regression was performed using the “logistf” package in R (version 4.4.2).

## 3 Results

### 3.1 Study population and baseline characteristics

**Figure 1:**
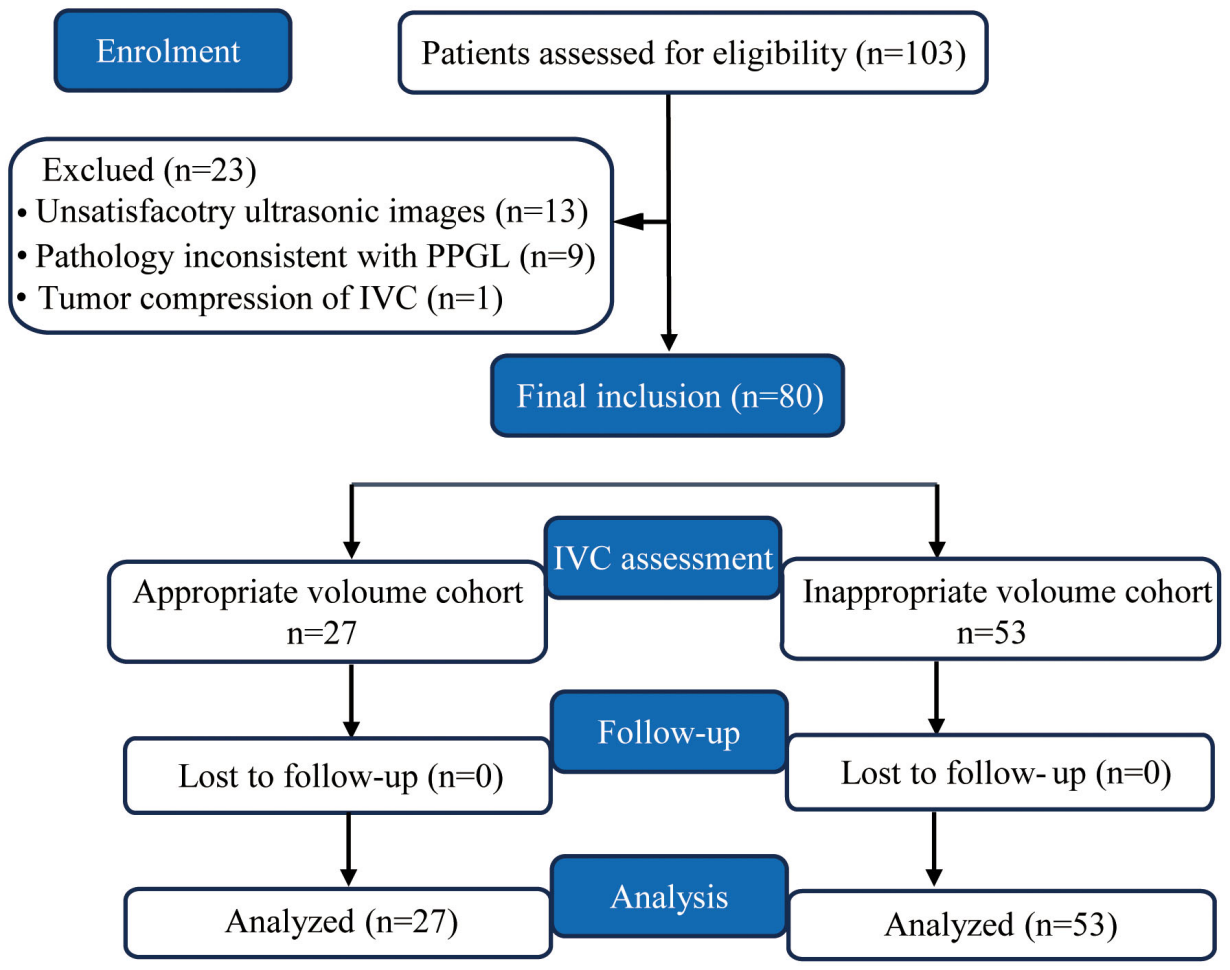
Patient flow chart.

Patient enrollment was terminated due to the expiration of project funding, prior to reaching the predetermined sample size. This was attributable to the slow recruitment rate for this rare disease. The final study sample comprised 80 patients (**Figure 1**), all patients completed 30 days follow-up, and there was no missing data. The data from 80 patients were analyzed. Based on preoperative IVC ultrasound assessment, 27 (33.7%) patients were categorized as having appropriate volume (AV cohort), while 53 (66.3%) were categorized as having inappropriate volume (IAV cohort). Patient characteristics for each cohort were summarized in **Table 1**.

**Table 1:** Patient characteristics.

Variables	Appropriate volume cohort	Inappropriate volume cohort	Overall
Number of patients	27 (33.7%)	53 (66.3%)	80 (100.0%)
Demographics			
Age (year)	48 ± 13	45 ± 13	45 ± 13
Male, n (%)	10 (37.0%)	27 (49.1%)	37 (46.3%)
Weight (kg)	67.9 ± 13.2	66.4 ± 13.5	66.6 ± 13.1
BMI (kg/m2)	24.7 ± 3.4	23.6 ± 3.6	24.0 ± 3.5
Clinical characteristics			
Tumor size^1^ (mm)	42 (25, 51)	37 (29, 55)	39 (27, 54)
Plasma metanephrine (nmol/L)	0.22 (0.13, 3.12)	0.55 (0.16, 2.38)	0.48 (0.14, 2.50)
Plasma normetanephrine (nmol/L)	2.96 (1.60, 10.38)	6.20 (2.01, 8.16)	4.18 (1.82, 9.49)
Plasma 3-metoxitiramine (nmol/L)	0.029 (0.017, 0.050)	0.033 (0.018, 0.047)	0.032 (0.018, 0.048)
ACCI	4 (3, 6)	4 (2, 5)	4 (2, 5)
ARISCAT score	31 (15, 38)	31 (15, 34)	31 (15, 37)
Preoperative α-blockade treatment			
Duration of treatment (day)	60 (30, 100)	60 (30, 89)	60 (30, 98)
Dosage (mg/d)	30 ± 14	26 ± 9	29 ± 12
Weight gain (kg)	2.4 ± 2.0	1.6 ± 3.0	1.9 ± 2.6
Decreased HCT (%)	2.2 ± 2.8	1.2 ± 3.8	1.5 ± 3.4
Supine SBP (mmHg)	126 ± 18	123 ± 15	124 ± 16
Supine HR (bpm)	80 ± 10	74 ± 11	76 ± 11
Upright SBP (mmHg)	104 ± 14	104 ± 22	104 ± 20
Upright HR (bpm)	99 ± 18	116 ± 113	111 ± 96

^1^Tumer size referred to the maximum diameter of the tumor measured on CT imaging. BMI, body mass index; ACCI, Age-adjusted Charlson Comorbidity Index; ARISCAT, Assess Respiratory Risk in Surgical Patients in Catalonia; HCT, hematocrit; SBP, systolic blood pressure; HR, heart rate.

### 3.2 Anesthesia and surgical management

All the patients received standardized anesthesia and surgical treatment as described in the Methods section. Key intraoperative parameters were presented in **Table 2**.

**Table 2:** Surgical and anesthesia management.

Variables	Appropriate volume cohort	Inappropriate volume cohort
Number of patients	27 (33.7%)	53 (66.3%)
Surgical duration (min)	122 (87, 160)	139 (87, 132)
Anesthesia duration (min)	197 (163, 241)	205 (161, 250)
Infusion volume^2^ (ml)	3000 (2500, 3600)	3100 (2550, 3650)
Operative blood loss (ml)	100 (50, 200)	50 (50, 75)
Urine output (ml)	300 (175, 425)	300 (200, 550)
Intraoperative dose of noradrenaline (ug/kg)	6.10 (2.50, 13.74)	7.45 (0.88, 33.88)
Intraoperative dose of adrenaline (ug/kg)	0 (0,0)	0 (0,0)
Intraoperative dose of phentolamine (mg)	1 (0, 4.00)	0 (0, 2)
Intraoperative dose of nitroprusside (ug/kg)	0 (0, 0)	0 (0, 0)
Intraoperative vasopressor^3^ use, n (%)	26 (96.3%)	43 (81.1%)
Intraoperative antihypertensive^4^ use, n (%)	20 (74.1%)	28 (52.8%)

^2^Infusion volume referred to the total volume of crystalloid and colloid solutions.

^3^Vasopressor referred to noradrenaline and adrenaline.

^4^Antihypertensive referred to phentolamine and nitroprusside.

### 3.3 Preoperative intravascular volume status

IVC ultrasound assessment identified inappropriate volume in 53 (66.3%) patients (51 with hypovolemia and 2 with hypervolemia) and appropriate volume in 27 (33.7%) patients (**Table 3**). Assessment of clinical parameters revealed that 37 (46.3%) patients exhibited orthostatic hypotension, 12 (15%) patients did not report nasal congestion, 14 (17.5%) patients had an upright SBP < 90 mmHg, and 30 (37.5%) patients did not demonstrate a decrease in HCT. The composite clinical parameter identified 63 (78.8%) patients with inappropriate volume status, whereas 17 (21.2%) patients with appropriate volume status. Agreement between IVC ultrasound and clinical parameters in assessing IV status was poor, as indicated by the weighted kappa values (**Table 3**).

**Table 3:** Agreement of preoperative intravascular volume measurements between clinical assessment and IVC ultrasound.

Variables	Appropriate volume cohort	Inappropriate volume cohort	Weighted Kappa	95% CI
Number of patients	27 (33.7%)	53 (66.3%)		
Orthostatic hypotension, n (%)	15 (55.6%)	22 (41.5%)	-0.123	-0.325, 0.079
Nasal congestion, n (%)	22 (81.5%)	46 (86.8%)	-0.039	-0.165, 0.088
Upright SBP <90mmHg, n (%)	5 (18.5%)	9 (17.0%)	-0.011	-0.143, 0.120
Decrease in HCT, n (%)	24 (88.9%)	34 (64.2%)	0.193	0.046, 0.340
Gain weight, n (%)	19 (70.4%)	31 (58.5%)	0.098	-0.083, 0.280
Composite clinical parameter^5^, n (%)	9 (33.3%)	8 (15.1%)	0.139	-0.079, 0.357

^5^A positive composite clinical parameter was defined as concordant evidence of appropriate volume status across all five clinical parameters. Specifically, the patient was free of orthostatic symptoms, maintained an upright SBP ≥ 90 mmHg, and exhibited nasal congestion, a decrease in HCT and an increase in body weight. Weighted kappa statistics were used.

SBP, systolic blood pressure; HCT, hematocrit.

### 3.4 Preoperative adverse outcomes in each cohort

In the AV cohort, 15 (55.6%) patients experienced intraoperative hemodynamic instability, and 11 (40.7%) patients experienced postoperative organ dysfunction. Overall, 21 (77.8%) patients experienced at least one PAO. In addition, 15 (55.6%) patients experienced one PAO, and 6 (28.6%) patients experienced two. In the IAV cohort, 22 (41.51%) patients experienced intraoperative hemodynamic instability, and 17 (32.1%) patients experienced postoperative organ dysfunction. Overall, 32 (60.4%) patients in the IAV cohort experienced at least one PAO. In addition, 19 (35.8%) patients experienced one PAO, 12 (22.6%) patients experienced two, and 1 (1.9%) patient experienced five. The severity of postoperative organ dysfunction was classified as Clavien-Dindo classification I or II (**Supplementary Table 2**).

### 3.5 Association between IVC ultrasound measurements and perioperative adverse outcomes

In both unadjusted and adjusted binary logistic regression models, we observed no association of IVC ultrasound assessed IV status and perioperative adverse outcome, intraoperative hemodynamic instability and postoperative organ dysfunction, respectively (**Table 4**).

**Table 4:** Associations between preoperative intravascular volume assessment and perioperative outcome .

Parameters	Comparisons between two cohorts	Perioperative adverse outcome	Intraoperative hemodynamic instability	Postoperative organ dysfunction
IVC ultrasound	AV cohort/IAV cohort, n/N (%)	21 (77.8%) / 32 (60.4%)	15 (55.56%) / 22 (41.51%)	11 (40.7%) / 17 (32.1%)
	Unadjusted OR (95% CI)	2.27 (0.29, 6.67)	1.75 (0.69, 4.55)	1.46 (0.56, 3.80)
	Adjusted OR^6^ (95% CI)	2.86 (0.84, 10.00)	1.45 (0.48, 4.35)	1.88 (0.61, 5.88)
Orthostatic hypotension	Cases exposed/ unexposed, n/N (%)	25 (67.6%) / 28 (65.1%)	19 (51.4%) / 18 (41.9%)	12 (32.4%) / 16 (37.2%)
	Unadjusted OR (95% CI)	1.12 (0.44, 2.83)	1.47 (0.61, 3.55)	0.81 (0.32, 2.04)
	Adjusted OR^6^ (95% CI)	1.15 (0.40, 3.29)	1.52 (0.52, 4.35)	0.55 (0.18, 1.67)
Upright SBP< 90mmHg	Cases exposed/ unexposed, n/N (%)	8 (57.1%) / 45 (68.2%)	3 (21.4%) / 34 (51.5%)	5 (35.7%) / 23 (34.8%)
	Unadjusted OR (95% CI)	0.62 (0.19, 2.02)	0.26 (0.07, 1.01)	1.04 (0.31, 3.47)
	Adjusted OR^6^ (95% CI)	0.66 (0.17, 2.63)	0.35 (0.08, 1.61)	1.06 (0.26, 4.26)
Gain weight	Cases exposed/ unexposed, n/N (%)	31 (62.0%) / 22 (73.3%)	22 (44.0%) / 15 (50.0%)	14 (28%) / 14 (46.7%)
	Unadjusted OR (95% CI)	0.59 (0.22, 1.59)	0.79 (0.32, 1.96)	0.44 (0.17, 1.15)
	Adjusted OR^6^ (95% CI)	0.40 (0.53, 4.85)	0.97 (0.32, 2.96)	0.43 (0.14, 1.27)
Decrease in HCT	Cases exposed/ unexposed, n/N (%)	39 (67.2%) / 14 (63.3%)	27 (46.6%) / 10 (45.5%)	20 (34.5%) / 8 (36.4%)
	Unadjusted OR (95% CI)	1.18 (0.42, 3.23)	1.04 (0.39, 2.78)	0.92 (0.33, 2.56)
	Adjusted OR^6^ (95% CI)	1.40 (0.45, 4.37)	1.11 (0.35, 3.60)	0.88 (0.27, 2.78)
Nasal congestion	Cases exposed/ unexposed, n/N (%)	44(64.7%)/9(75.0%)	31(45.6%)/6(50.0%)	23(33.8%)/5(41.7%)
	Unadjusted OR (95% CI)	0.61 (0.15, 2.5)	0.84 (0.21, 2.86)	0.71 (0.20, 2.50)
	Adjusted OR^6^ (95% CI)	0.65 (0.14, 3.06)	0.66 (0.15, 2.86)	0.45 (0.10, 1.98)

Binary logistic regression model was used. ^6^ Odds ratio adjusted for tumor size, plasma metanephrine, plasma normetanephrine, sex, age, age-adjusted Charlson Comorbidity Index, and ARSCAT score. AV, appropriate volume; IAV, inappropriate volume.

### 3.6 Association between clinical parameters and perioperative adverse outcome

Neither unadjusted nor adjusted binary logistic regression models demonstrated any significant association between the clinical parameters estimating preoperative IV and perioperative outcomes (**Table 4**).

### 3.7 Performance of preoperative intravascular volume assessments in predicting perioperative adverse outcomes

This study evaluated the performance of preoperative IVC ultrasound for predicting PAOs, comparing it to that of clinical parameters. The areas under the ROC curve (AUC) were calculated and compared (**Table 5**, **Supplementary Figure 1**). ROC analysis revealed modest discriminatory performance for dichotomized IVC parameters, with AUCs ranging from 0.54 to 0.59 for the perioperative outcome (**Table 5**). Notably, these values were comparable to or lower than those of traditional clinical parameters and the composite clinical parameters (**Table 5**). The combined model incorporating both IVC ultrasound and clinical parameters achieved the highest discriminatory performance (AUC 0.64–0.67), though the incremental gain attributable specifically to IVC ultrasound was marginal. However, no significant differences in AUC values were observed (**Supplementary Table 3**). These findings suggested that while IVC ultrasound provided physiological information regarding IV status, its standalone predictive value in this cohort did not exceed that of established clinical assessments.

**Table 5:** ROC curve areas for preoperative intravascular volume estimation.

Parameters	Perioperative adverse outcome	Intraoperative hemodynamic instability	Postoperative organ dysfunction
IVC ultrasound	0.59 (0.48, 0.69)	0.56 (0.46, 0.67)	0.54 (0.43, 0.65)
Orthostatic hypotension symptoms	0.51 (0.40, 0.63)	0.55 (0.44, 0.66)	0.53 (0.41, 0.64)
Upright SBP< 90mmHg	0.54 (0.44, 0.63)	0.59 (0.51, 0.67)	0.50 (0.41, 0.59)
Gain weight	0.60 (0.49, 0.71)	0.53 (0.42, 0.64)	0.60 (0.48, 0.71)
Decrease in HCT	0.52 (0.41, 0.62)	0.50 (0.41, 0.60)	0.51 (0.40, 0.61)
Nasal congestion	0.530 (0.45, 0.61)	0.51 (0.43, 0.59)	0.52 (0.44, 0.61)
Composite clinical parameters^7^	0.61 (0.48, 0.75)	0.66 (0.54, 0.78)	0.61 (0.49, 0.74)
Composite all parameters^8^	0.67 (0.54, 0.79)	0.67 (0.55, 0.79)	0.64 (0.52, 0.76)

Values were expressed as point estimate with 95% CI.

^7^Composite clinical parameter was defined as the integrated score derived from five clinical parameters.

^8^Composite all parameters combined five clinical parameters with the IVC ultrasound assessments.

### 3.8 Sensitivity analyses

Firth logistic regression was applied to investigate the preoperative IV assessment (both by IVC measurements and clinical parameters) and perioperative outcome. It yielded consistent results (**Supplementary Table 3**).

For the choice of IVC ultrasound measurement cut-off values, likelihood-ratio tests revealed no evidence of non-linearity association between IVC ultrasound measurements and PAO (**Supplementary Figure 2**). It indicated that the absence of an association was unlikely to be explained by the choice of IVC ultrasound measurement cut-off values.

Restricted cubic spline analysis with 3 knots placed at the 10th, 50th, and 90th percentiles was used to examine potential nonlinear associations between preoperative IVC parameters and PAO. For IVCmax, the analysis revealed a monotonic association with the predicted probability of PAO (**Supplementary Figure 2**). The likelihood ratio test for nonlinearity was not significant (χ² =0.009, p = 0.92, **Supplementary Table 5**), suggesting that the association was adequately captured by a linear relationship. Similar linear trends were observed for IVCmin (χ² =0.002, p = 0.96, **Supplementary Table 5**) and cIVC (χ² =0.078, p = 0.78, **Supplementary Table 5**).The absence of significant nonlinearity suggested that clinically established cut-off values for IVC parameters may not substantially alter risk stratification.

Sensitivity analysis with dichotomous variables as continuous variables using Firth logistic regression still yielded consistent results (**Supplementary Table 6**, **S7**). Neither IVC measurements (IVC_max_, IVC_min_, and cIVC) and clinical parameters failed to show independently association with three perioperative outcomes.

Poisson regression showed that patients in AV group had an incidence rate ratio of 1.10 (95% CI, 0.68 to 1.76) for having PAO compared with those in IAV group, which was not statistically different.

## 4 Discussion

This prospective, single-center, observational cohort study is the first to characterize IVC ultrasound-assessed IV status after prolonged preoperative α-blockade and volume expansion in patients scheduled for catecholamine-secreting PPGL resection. The study population carried a heavy comorbidity burden (median ACCI 4), a threshold previously associated with moderate-to-high risk for major perioperative morbidity. Consequently, preoperative optimization was protracted (median 60 days). Despite this extended preparation, 66.3% of patients still exhibited inappropriate volume status, almost exclusively hypovolemia (96.2%). Neither IVC ultrasound assessment nor clinical parameters assessment of IV was associated with PAOs. Preoperative IVC ultrasound did not demonstrate incremental discriminatory value beyond traditional clinical parameters. We performed a series of sensitivity analyses. First, we reanalyzed the data using Firth logistic regression. The results were consistent with those obtained from the primary analysis (logistic regression), indicating that our conclusions were not sensitive to the choice of regression method. Second, we modeled IVC measurements (IVC_max_, IVC_min_, and cIVC) as continuous variables using restricted cubic splines. The likelihood ratio tests for nonlinearity were not significant for all three IVC parameters, suggesting that the associations between preoperative IV assessment and PAOs were adequately captured by linear relationships. These findings mitigated concerns that the choice of cut-off values may have influenced our primary results. Third, we repeated the Firth logistic regression analysis using continuous rather than dichotomized variables. Again, the results were consistent with the primary analysis, further supporting the robustness of our conclusions across different variable specifications and modeling approaches. Collectively, these sensitivity analyses suggested that the observed absence of association between preoperative IV status and perioperative outcomes were unlikely to be artifacts of statistical methodology or arbitrary cut-off selection. Meanwhile, with respect to the number of complications, our data failed to show any statistically significant difference between the two cohorts (IAV and AV).

The ROC analyses showed that IVC ultrasound achieved AUCs of 0.54–0.59, comparable to or lower than traditional clinical parameters (0.61–0.66) and their composite scores (0.61–0.67). This suggests that standalone IVC measurements offered limited incremental classification value beyond clinical assessments in this medically optimized cohort, although both modalities demonstrated modest discriminatory performance. Of note, these findings should be interpreted as inconclusive rather than evidence of absent predictive value. A critical consideration in interpreting the near-random AUCs was the potential for meticulous intraoperative hemodynamic management to attenuate preoperative risk signals. If GDFT and vasoactive management successfully compensated for preoperative volume abnormalities, then near-random discrimination would not necessarily indicate that preoperative volume status lacked prognostic value. Rather, it may reflect effective intraoperative neutralization of volume-related risk before manifestation in measured outcomes.

Our center performs the largest volume of PPGL resections in China and employs a structured multidisciplinary protocol (endocrinology, urology, anesthesiology and critical care medicine). At this high-volume, highly experienced center, we speculated that the impacts of preoperative inappropriate volume status determined by IVC ultrasound may be compensated by the GDFT as well as experienced hemodynamic management during and post-surgery. Advanced techniques such as pulse contour analysis, transesophageal Doppler, and TEE enable real-time assessment of blood volume, cardiac systolic function, and vascular resistance during surgery (15, 16), guiding appropriate interventions and improving patient outcomes. Thus, the advantage of IVC ultrasound assessment of preoperative volume might be overridden. However, it is important to contextualize these findings within the broader landscape of perioperative care. While our results suggested that preoperative IVC ultrasound had limited independent predictive value in high-volume academic centers with meticulous intraoperative GDFT protocols, this may not be generalizable to all clinical settings. In low-volume or non-academic centers where real-time advanced hemodynamic monitoring (e.g., transesophageal Doppler, TEE, pulse contour analysis) is unavailable, preoperative IVC assessment may retain great clinical utility. In such settings, IVC ultrasound could serve as a readily available, non-invasive screening tool to identify patients with significant IV deficits who might benefit from more aggressive preoperative optimization or referral to higher-level care. Furthermore, the simplicity and portability of point-of-care ultrasound make it particularly valuable in resource-limited environments where sophisticated intraoperative monitoring cannot be routinely implemented. Future studies should validate the predictive performance of preoperative IVC parameters in diverse clinical settings with varying levels of intraoperative monitoring capability to better define its role across the spectrum of perioperative care. Additionally, enrolled patients presented a high burden of comorbidity, thus leading to a longer duration for preoperative medication and treatment. This extended preparation period may have substantially reduced the incidence of severe IV depletion, thereby lowering the baseline risk of PAOs. Consequently, the potential advantage of preoperative IVC ultrasound-guided volume assessment in predicting poor outcomes may have been attenuated.

Although the enrolled patients exhibited heterogeneous preoperative volume status, heart rate and blood pressure control in both supine and upright positions were well controlled prior to surgery. For these patients, other factors, such as age, tumor size, circulating catecholamine concentrations, and comorbidities might mainly contribute to PAOs rather than preoperative IV. Although our data did to support the routine preoperative point-of-care IVC ultrasound for PPGL resection patients with a long duration of preoperative preparation, the question remains open in centers with lower surgical volumes or in cohorts receiving abbreviated or no α-blockade.

For the first time, our data showed a poor agreement of IVC ultrasound and clinical parameters for assessing the preoperative IV. Given the established correlation between IVC measurements and RA pressure, our finding implied that conventional clinical parameters may have limited accuracy in estimating static IV. However, poor agreement did not indicate that clinical parameters were universally invalid. Rather, their utility may depend on the intended purpose: clinical parameters chosen to reflect static IV status may have limited value, whereas those capturing tissue perfusion, organ function, or hemodynamic compensation may retain clinical significance.

For the first time our data demonstrated that traditional clinical parameters used to estimate volume restoration failed to show any association with the PAO, including orthostatic hypotension, weight gain, decreased HCT, nasal congestion, and upright blood pressure < 90 mmHg. While intraoperative fluid strategies might again confound this relationship, our findings cautioned against declaring inadequate preparation solely based on these signs after prolonged α-blockade.

There are several limitations. First, the relatively small sample size had limited our power to detect statistically significant differences. A power analysis suggested a target sample size of 364, however, the rarity of PPGLs constrained enrollment within the funded research period. After final analysis, data from 80 patients showed that it was unlikely to suggest that patients with appropriate volume status assessed by IVC ultrasound had fewer PAO. We acknowledge that the relatively small sample size (n = 80) and low event rate limit the precision of our effect estimates, as reflected by the wide confidence intervals. These findings should be interpreted as hypothesis-generating rather than definitive. Meanwhile, due to the limited sample size, we were unable to test for interaction effects between variables. Consequently, potential effect modification cannot be ruled out. Future studies with larger cohorts are warranted to explore these interactions and confirm these associations. Second, due to high burden of comorbidity, the duration of preoperative α-blockade (median 60 days) for patients was extended. It is unclear whether varying durations of α-blockade influence outcomes of present study. Therefore, further prospective research is needed to for populations undergoing shorter or no α-blockade. Third, intraoperative sustained hypertension and hypotension likely have distinct mechanisms. Separate component analyses were limited by low event counts. We acknowledged this as a limitation and recommended that future adequately powered studies distinguish hypertensive and hypotensive events to clarify specific associations with preoperative volume status. Similarly, future adequately powered studies should also examine the associations between preoperative volume status and individual organ-specific postoperative dysfunctions. Last, as a single-center observational cohort study, our results were susceptible to unmeasured and residual confounding. Unmeasured patient factors, surgical and anesthetic techniques, may all influence the outcomes.

## 5 Conclusion

In this study with a sample size of 80 patients, neither IVC ultrasound nor conventional clinical parameters of IV status were statistically associated with perioperative adverse outcome in patients undergoing prolonged preoperative preparation for PPGL resection. The utility of point-of-care IVC ultrasound in guiding preoperative volume optimization for these patients merits further exploration.

## Data Availability

The raw data supporting the conclusions of this article will be made available by the authors, without undue reservation.
