# Supplementary material for: Preoperative inferior vena cava ultrasound for volume optimization in resection of functioning pheochromocytoma and paraganglioma: association with perioperative adverse outcomes in a prospective observational cohort
Source: Front Endocrinol (Lausanne). 2026 Jul 23;17:1844538. doi: 10.3389/fendo.2026.1844538 (PMC13442004; doi:10.3389/fendo.2026.1844538)
Supplement: Supplementary file 1 [file SupplementaryFile1.docx]

Supplementary Material

# 1 Supplementary Figures and Tables

For more information on Supplementary Material and for details on the different file types accepted, please see [here](https://www.frontiersin.org/guidelines/author-guidelines#supplementary-material).

## 1.1 Supplementary Figures

**Figure S1.** ROC curves for perioperative outcome prediction. (A) ROC curves for predicting perioperative adverse outcome. (B) ROC curves for predicting intraoperative hemodynamic instability. (C) ROC curves for predicting postoperative organ dysfunction. AUCs did not differ significantly among IVC, CCP and CAP for three endpoints. ROC, receiver-operating-characteristic curve; AUC, area under curve; IVC, inferior vena cava; CCP, composite of clinical parameters, which was defined as the integrated score derived from five clinical parameters; CAP, composite of all parameters, which combined five clinical parameters with the IVC ultrasound assessments.

**Figure S2.** Restricted cubic spline analysis of the associations between preoperative IVC ultrasound measurements and the predicted probability of perioperative adverse outcome. Predicted probabilities of adverse events (solid blue lines) and 95% confidence intervals (shaded areas) were derived from logistic regression models with restricted cubic splines (3 knots placed at the 10th, 50th, and 90th percentiles of each exposure variable). The reference value was set at the median of each variable (vertical red line). Vertical dotted lines indicated the positions of the knots. The horizontal dashed line denoted the average predicted probability of adverse outcomes in the study cohort. P values for nonlinearity were 0.92, 0.96, and 0.78 for IVC_max_, IVC_min_, and cIVC, respectively.

## 1.2 Supplementary Tables

**Table S1.** STROBE Statement—Checklist of items that should be included in reports of ***cohort studies***

|  | | Item No | Recommendation | | Page No |
| --- | --- | --- | --- | --- | --- |
| **Title and abstract** | | 1 | (*a*) Indicate the study’s design with a commonly used term in the title or the abstract | | 1 |
|  |  |  | (*b*) Provide in the abstract an informative and balanced summary of what was done and what was found | | 1-2 |
| Introduction | | | | | |
| Background/rationale | | 2 | Explain the scientific background and rationale for the investigation being reported | | 2 |
| Objectives | | 3 | State specific objectives, including any prespecified hypotheses | | 2 |
| Methods | | | | | |
| Study design | | 4 | Present key elements of study design early in the paper | | 2-3 |
| Setting | | 5 | Describe the setting, locations, and relevant dates, including periods of recruitment, exposure, follow-up, and data collection | | 2-3 |
| Participants | | 6 | (*a*) Give the eligibility criteria, and the sources and methods of selection of participants. Describe methods of follow-up | | 3 |
|  |  |  | (*b*) For matched studies, give matching criteria and number of exposed and unexposed | |  |
| Variables | | 7 | Clearly define all outcomes, exposures, predictors, potential confounders, and effect modifiers. Give diagnostic criteria, if applicable | | 3-4 |
| Data sources/ measurement | | 8* | For each variable of interest, give sources of data and details of methods of assessment (measurement). Describe comparability of assessment methods if there is more than one group | | 3-4 |
| Bias | | 9 | Describe any efforts to address potential sources of bias | | 3-4 |
| Study size | | 10 | Explain how the study size was arrived at | | 6 |
| Quantitative variables | | 11 | Explain how quantitative variables were handled in the analyses. If applicable, describe which groupings were chosen and why | | 3-4 |
| Statistical methods | | 12 | (*a*) Describe all statistical methods, including those used to control for confounding | | 5 |
|  |  |  | (*b*) Describe any methods used to examine subgroups and interactions | | 5 |
|  |  |  | (*c*) Explain how missing data were addressed | | 5 |
|  |  |  | (*d*) If applicable, explain how loss to follow-up was addressed | | 5 |
|  |  |  | (*e*) Describe any sensitivity analyses | | 5 |
| Results | | | | |  |
| Participants | | 13* | (a) Report numbers of individuals at each stage of study—eg numbers potentially eligible, examined for eligibility, confirmed eligible, included in the study, completing follow-up, and analysed | | 6 |
|  |  |  | (b) Give reasons for non-participation at each stage | | 6 |
|  |  |  | (c) Consider use of a flow diagram | | Figure1 |
| Descriptive data | | 14* | (a) Give characteristics of study participants (eg demographic, clinical, social) and information on exposures and potential confounders | | 6 |
|  |  |  | (b) Indicate number of participants with missing data for each variable of interest | | 6 |
|  |  |  | (c) Summarise follow-up time (eg, average and total amount) | | 6 |
| Outcome data | | 15* | Report numbers of outcome events or summary measures over time | | 6-7 |
| Main results | | 16 | (a) Give unadjusted estimates and, if applicable, confounder-adjusted estimates and their precision (eg, 95% confidence interval). Make clear which confounders were adjusted for and why they were included | | 6 |
|  | |  | (b) Report category boundaries when continuous variables were categorized | | 7 |
|  | |  | (c) If relevant, consider translating estimates of relative risk into absolute risk for a meaningful time period | | Not applicable |
| Other analyses | | 17 | Report other analyses done—eg analyses of subgroups and interactions, and sensitivity analyses | | 7 |
| Discussion | | | | | |
| Key results | 18 | Summarise key results with reference to study objectives | | 7 | |
| Limitations | 19 | Discuss limitations of the study, taking into account sources of potential bias or imprecision. Discuss both direction and magnitude of any potential bias | | 8 | |
| Interpretation | 20 | Give a cautious overall interpretation of results considering objectives, limitations, multiplicity of analyses, results from similar studies, and other relevant evidence | | 7-8 | |
| Generalisability | 21 | Discuss the generalisability (external validity) of the study results | | 8 | |
| Other information | | | | | |
| Funding | 22 | Give the source of funding and the role of the funders for the present study and, if applicable, for the original study on which the present article is based | | 9 | |

*Give information separately for exposed and unexposed groups.

**Note:** An Explanation and Elaboration article discusses each checklist item and gives methodological background and published examples of transparent reporting. The STROBE checklist is best used in conjunction with this article (freely available on the Web sites of PLoS Medicine at http://www.plosmedicine.org/, Annals of Internal Medicine at http://www.annals.org/, and Epidemiology at http://www.epidem.com/). Information on the STROBE Initiative is available at http://www.strobe-statement.org.

**Table S2.** Occurrence and severity of postoperative organ dysfunction by Clavien-Dindo classification

| Postoperative organ dysfunction | Total number | Severity of postoperative complications | | | | | | |
| --- | --- | --- | --- | --- | --- | --- | --- | --- |
|  |  | I | Ⅱ | Ⅲ_a_ | Ⅲ_b_ | Ⅳ_a_ | Ⅳ_b_ | Ⅴ |
| Pulmonary |  |  |  |  |  |  |  |  |
| Atelectasis^a^ | 0 | - | - | - | - | - | - | - |
| Hepatic |  |  |  |  |  |  |  |  |
| Elevated TBil^b^ | 14 | 12 | 2 | - | - | - | - | - |
| Elevated ALT^b^ | 6 | 6 | - | - | - | - | - | - |
| Renal |  |  |  |  |  |  |  |  |
| Elevated Cr^b^ | 4 | 4 | - | - | - | - | - | - |
| Vascular event | - | - | - | - | - | - | - | - |
| Elevated hs-cTnI^b^ | 6 | 6 | - | - | - | - | - | - |
| New onset arrhythmia^c^ | 0 | - | - | - | - | - | - | - |
| Surgical site infection | 0 | - | - | - | - | - | - | - |

Data were presented as numbers

^a^ confirmed by chest X-ray.

^b^ confirmed by blood test.

^c^ confirmed by electrocardiogram.

TB, total bilirubin; ALT, alanine aminotransferase; Cr, creatinine; high-sensitivity cardiac troponin, hs-cTnI

**Table S3.** Comparison of areas under the ROC curves for perioperative outcome prediction

|  | Perioperative adverse outcome | | Intraoperative  hemodynamic  instability | | Postoperative  organ dysfunction | |
| --- | --- | --- | --- | --- | --- | --- |
|  | 95% CI | P | 95% CI | P | 95% CI | P |
| AUC_OH_ - AUC_IVC_ | -0.22, 0.07 | 0.31 | -0.16, 0.13 | 0.83 | -0.15, 0.18 | 0.84 |
| AUC_USBP_ - AUC_IVC_ | -0.20, 0.09 | 0.49 | -0.11, 0.16 | 0.73 | -0.19, 0.11 | 0.60 |
| AUC_GW_ - AUC_IVC_ | -0.19, 0.14 | 0.74 | -0.20, 0.13 | 0.67 | -0.12, 0.23 | 0.54 |
| AUC_HCT_ - AUC_IVC_ | -0.20, 0.05 | 0.26 | -0.19, 0.07 | 0.36 | -0.21, 0.15 | 0.71 |
| AUC_NC_ - AUC_IVC_ | -0.18, 0.07 | 0.37 | -0.18, 0.08 | 0.43 | -0.16, 0.12 | 0.77 |
| AUC_CCP_ - AUC_IVC_ | -0.14, 0.20 | 0.76 | -0.06, 0.26 | 0.22 | -0.11, 0.25 | 0.44 |
| AUC _CAP_ - AUC_IVC_ | -0.02, 0.18 | 0.11 | -0.02, 0.23 | 0.11 | -0.04, 0.24 | 0.18 |

OH, orthostatic hypotension; IVC, IVC ultrasound; USBP, Upright SBP< 90mmHg; GW, gain weight; HCT, decrease in HCT; NC, nasal congestion; CCP, composite of clinical parameters, which was defined as the integrated score derived from five clinical parameters.; CAP, composite of all parameters, which combined five clinical parameters with the IVC ultrasound assessments.

**Table S4.** Associations between preoperative intravascular volume assessment and perioperative outcomes using Firth logistic regression

|  |  | Perioperative adverse outcome | Intraoperative hemodynamic instability | Postoperative organ dysfunction |
| --- | --- | --- | --- | --- |
| IVC ultrasound | AV cohort/IAV cohort, n/N (%) | 21 (77.8%) / 32 (60.4%) | 15 (55.56%) / 22 (41.51%) | 11 (40.7%) / 17 (32.1%) |
|  | Unadjusted OR (95% CI) | 1.67 (0.81, 6.67) | 1.74 (0.69, 4.42) | 1.45 (0.56, 3.70) |
|  | Adjusted OR^1^  (95% CI) | 2.56 (0.85, 9.09) | 1.45 (0.50, 4.18) | 1.79 (0.65, 5.00) |
| Orthostatic hypotension | Cases exposed/  unexposed, n/N (%) | 25 (67.6%) / 28 (65.1%) | 19 (51.4%) / 18 (41.9%) | 12 (32.4%) / 16 (37.2%) |
|  | Unadjusted OR (95% CI) | 1.06 (0.40, 2.70) | 1.09 (0.44, 2.71) | 0.98 (0.37, 2.48) |
|  | Adjusted OR^1^  (95% CI) | 1.03 (0.34, 3.03) | 1.10 (0.37, 3.22) | 0.77 (0.25, 2.20) |
| Upright SBP< 90mmHg | Cases exposed/  unexposed, n/N (%) | 8 (57.1%) / 45 (68.2%) | 3 (21.4%) / 34 (51.5%) | 5 (35.7%) / 23 (34.8%) |
|  | Unadjusted OR (95% CI) | 0.62 (0.20, 2.00) | 0.29 (0.07, 0.96) | 1.07 (0.32, 3.36) |
|  | Adjusted OR^1^  (95% CI) | 0.71 (0.19, 2.67) | 0.42 (0.09, 1.60) | 1.03 (0.40, 4.00) |
| Gain weight | Cases exposed/  unexposed, n/N (%) | 31 (62.0%) / 22 (73.3%) | 22 (44.0%) / 15 (50.0%) | 14 (28%) / 14 (46.7%) |
|  | Unadjusted OR (95% CI) | 0.61 (0.22, 1.59) | 0.79 (0.32, 1.93) | 0.45 (0.18, 1.14) |
|  | Adjusted OR^1^  (95% CI) | 0.68 (0.23, 1.92) | 0.98 (0.35, 2.70) | 0.47 (0.17, 1.31) |
| Decrease in HCT | Cases exposed/  unexposed, n/N (%) | 39 (67.2%) / 14 (63.3%) | 27 (46.6%) / 10 (45.5%) | 20 (34.5%) / 8 (36.4%) |
|  | Unadjusted OR (95% CI) | 1.19 (0.42, 3.23) | 1.04 (0.39, 2.77) | 0.91 (0.34, 2.54) |
|  | Adjusted OR^1^  (95% CI) | 1.39 (0.47, 4.17) | 1.12 (0.37, 3.47) | 0.96 (0.91, 1.01) |
| Nasal congestion | Cases exposed/  unexposed, n/N (%) | 44(64.7%)/9(75.0%) | 31(45.6%)/6(50.0%) | 23(33.8%)/5(41.7%) |
|  | Unadjusted OR (95% CI) | 0.67 (0.16, 2.33) | 0.84 (0.25, 2.81) | 0.70 (0.21, 2.47) |
|  | Adjusted OR^1^  (95% CI) | 0.70 (0.15, 2.78) | 1.43 (0.36, 6.01) | 0.96 (0.91, 1.00) |

Firth logistic regression model was used. ^1^ Odds ratio adjusted for tumor size, plasma metanephrine, plasma normetanephrine, sex, age, age-adjusted Charlson Comorbidity Index, and ARSCAT score. AV, appropriate volume; IAV, inappropriate volume; IVC, inferior vena cava; SBP, systolic blood pressure; HCT, hematocrit.

**Table S5.** Restricted cubic spline analysis of the associations between preoperative IVC ultrasound measurements and the predicted probability of perioperative adverse events

| Variable | Knots (10th/50th/90th) | Reference | LR χ² for nonlinearity | p value |
| --- | --- | --- | --- | --- |
| IVC_max_ | 1.07, 1.50, 1.97 | 1.50 | 0.009 | 0.92 |
| IVC_min_ | 0.85, 1.20, 1.60 | 0.59 | 0.002 | 0.96 |
| cIVC | 15.0, 30.0, 45.0 | 58.61 | 0.078 | 0.78 |

IVC, inferior vena cava; cIVC, IVC collapsibility index; LR, likelihood ratio. P values for nonlinearity were derived from likelihood ratio tests comparing linear models with restricted cubic spline models.

**Table S6.** Associations between preoperative intravascular volume assessment and perioperative outcomes

using continuous variables

| Variable | | Perioperative adverse outcome | | Intraoperative hemodynamic instability | | Postoperative organ dysfunction | |
| --- | --- | --- | --- | --- | --- | --- | --- |
|  |  | OR  (95% CI) | P  value | OR  (95% CI) | P  value | OR  (95% CI) | P value |
| IVC_max_  (per mm) | Unadjusted | 1.02  (0.89, 1.18) | 0.77 | 1.01  (0.89, 1.16) | 0.84 | 1.03  (0.90, 1.19) | 0.64 |
|  | Adjusted ^1^ | 0.99  (0.84, 1.15) | 0.86 | 0.97  (0.83, 1.13) | 0.69 | 1.00  (0.87, 1.16) | 0.95 |
| IVC_min_  (per mm) | Unadjusted | 1.10  (0.95, 1.29) | 0.23 | 1.03  (0.91, 1.18) | 0.62 | 1.10  (0.96, 1.26) | 0.19 |
|  | Adjusted ^1^ | 1.09  (0.94, 1.29) | 0.29 | 0.99  (0.85, 1.15) | 0.89 | 1.13  (0.97, 1.31) | 0.12 |
| cIVC  (per 1%) | Unadjusted | 0.98  (0.95, 1.01) | 0.15 | 0.99  (0.96, 1.02) | 0.49 | 0.98  (0.96, 1.01) | 0.21 |
|  | Adjusted ^1^ | 0.98  (0.94, 1.01) | 0.15 | 1.00  (0.97, 1.03) | 0.87 | 0.97  (0.94, 1.00) | 0.07 |
| Upright SBP  (per mmHg) | Unadjusted | 1.01  (0.98, 1.04) | 0.65 | 1.04  (1.00, 1.07) | 0.03 | 0.99  (0.96, 1.02) | 0.47 |
|  | Adjusted ^1^ | 0.99  (0.82, 1.21) | 0.95 | 1.04  (1.00, 1.08) | 0.06 | 0.98  (0.95, 1.01) | 0.24 |
| Gain weight  (per kg) | Unadjusted | 1.00  (0.84, 1.21) | 0.97 | 0.99  (0.83, 1.17) | 0.87 | 1.00  (0.83, 1.19) | 1.00 |
|  | Adjusted ^1^ | 1.03  (0.84, 1.25) | 0.76 | 1.03  (0.84, 1.25) | 0.76 | 0.95  (0.79, 1.16) | 0.63 |
| Decrease in HCT  (per 1%) | Unadjusted | 1.03  (0.90, 1.18) | 0.68 | 1.04  (0.92, 1.19) | 0.51 | 1.01  (0.88, 1.15) | 0.93 |
|  | Adjusted ^1^ | 1.03  (0.90, 1.19) | 0.65 | 1.04  (0.90, 1.21) | 0.57 | 1.03  (0.89, 1.19) | 0.72 |

Firth regression model was used. ^1^ Odds ratio (OR) adjusted for tumor size, plasma metanephrine, plasma normetanephrine, sex, age, age-adjusted Charlson Comorbidity Index, and ARSCAT score. CI, confidence interval; IVC, inferior vena cava; SBP, systolic blood pressure; HCT, hematocrit.

**Table S7**. Independent associations of IVC measurements with perioperative outcomes

by Firth logistic Regression

|  | Perioperative adverse outcome | | Intraoperative hemodynamic instability | | Postoperative organ dysfunction | |
| --- | --- | --- | --- | --- | --- | --- |
| Variable | OR  (95% CI) | P  value | OR  (95% CI) | P  value | OR  (95% CI) | P  value |
| IVCmax  (per mm) | 0.98  (0.63, 1.57) | 0.92 | 1.06  (0.70, 1.67) | 0.79 | 0.89  (0.57, 1.34) | 0.59 |
| IVCmin  (per mm) | 0.95  (0.32, 2.42) | 0.92 | 0.82  (0.33, 1.92) | 0.65 | 1.22  (0.54, 3.03) | 0.63 |
| cIVC  (per 1%) | 0.97  (0.81, 1.12) | 0.68 | 0.97  (0.84, 1.11) | 0.62 | 1.00  (0.88, 1.16) | 0.96 |
| Age (year) | 1.00  (0.93, 1.05) | 0.90 | 1.02  (0.96, 1.07) | 0.57 | 0.97  (0.92, 1.02) | 0.24 |
| Sex | 2.12  (0.70, 6.94) | 0.18 | 0.90  (0.29, 2.76) | 0.85 | 3.53  (1.26, 10.81) | 0.02 |
| Tumor size (mm) | 1.02  (0.99, 1.06) | 0.18 | 1.03  (1.00, 1.07) | 0.02 | 0.99  (0.97, 1.01) | 0.28 |
| Metanephrine  (nmol/L) | 0.99  (0.98, 1.01) | 0.31 | 0.99  (0.97, 1.00) | 0.11 | 1.00  (0.96, 1.01) | 0.88 |
| Normetanephrine  (nmol/L) | 1.00  (1.00, 1.00) | 0.49 | 1.00  (1.00, 1.00) | 0.23 | 1.00  (1.00, 1.00) | 0.74 |
| ACCI | 1.41  (0.90, 2.68) | 0.16 | 1.54  (1.04, 2.56) | 0.03 | 1.35  (0.96, 1.98) | 0.08 |
| ARISCAT | 0.99  (0.93, 1.04) | 0.61 | 0.96  (0.90, 1.01) | 0.13 | 1.04  (0.99, 1.09) | 0.16 |

Firth logistic regression analyses were performed to evaluate the independent associations of inferior vena cava measurements with perioperative outcomes. IVC, inferior vena cava; OR, odds ratio; CI, confidence interval; ACCI, age-adjusted Charlson Comorbidity Index; ARISCAT, Assess Respiratory Risk in Surgical Patients in Catalonia. Sex was presented as binary variable, while the others as continuous variable.
